# Direct traction MR imaging of the wrist: practical experience

**DOI:** 10.1007/s00256-024-04842-w

**Published:** 2024-12-04

**Authors:** Miaoru Zhang, Stefanie W. Y. Yip, Su Wu, David K. W. Yeung, James F. Griffith

**Affiliations:** Department of Imaging and Interventional Radiology, Prince of Wales Hospital, The Chinese University of Hong Kong, 30-32 Ngan Shing Street, Shatin, New Territories, Hong Kong, SAR China

**Keywords:** Magnetic resonance imaging, Wrist, Joint distraction, Cartilage, Intrinsic ligaments, Triangular fibrocartilage complex

## Abstract

**Objective:**

To study the effect of direct wrist traction on patient pain and joint distraction on MRI.

**Materials and methods:**

291 patients (109 males, 182 females; mean age, 45.8 years) who underwent wrist MRI between November 2019 and September 2024 were studied (152 patients with traction, 139 patients without traction). All patients completed a questionnaire assessing wrist pain scores before, during, and ten minutes after MRI examination. Joint space width and cartilage visibility of the radiocarpal and intercarpal joints were assessed. For patients with arthroscopy within one year after MRI, diagnostic accuracy of intrinsic ligament and triangular fibrocartilage complex tears was assessed.

**Results:**

Fifty-seven (38%) of 152 traction patients had an average increase of 1 point (range, 0 ~ 2) in wrist pain following traction compared to 24 (17%) of 139 non-traction patients (*p* = 0.085). 44% traction patients and 38% non-traction patients reported non-wrist pain (mainly shoulder, neck), with no inter-group difference in location, prevalence, or pain score (all *p* values > 0.05). Average joint space width was 0.6 mm wider in the traction group (*p* < 0.001). On average, eighty-five (60%) of 141 traction patients had ‘moderate’ or ‘good’ articular cartilage visibility compared to 22 (17%) of 126 non-traction patients (*p* < 0.001). Traction tended to increase diagnostic accuracy for intrinsic ligament tear, though it did not reach statistical significance (*p* = 0.136).

**Conclusion:**

Compared to wrist MRI without traction, traction increases joint space width and improves cartilage visibility, though with a slight increase in wrist pain.

**Supplementary Information:**

The online version contains supplementary material available at 10.1007/s00256-024-04842-w.

## Introduction

MRI is the primary imaging modality used to identify wrist pathology, including articular cartilage, intrinsic ligament, and triangular fibrocartilage complex (TFCC) injury. The small wrist joint space and close apposition of structures can limit the visibility of the articular cartilage, intrinsic ligaments, and TFCC on standard MRI [1]. Traction applied during MRI effectively distracts the wrist joint space and improves the visibility of articular cartilage, intrinsic ligament, and TFCC [2, 3]. The accuracy of wrist MR arthrography with traction in diagnosing lunotriquetral and scapholunate ligament injuries, as well as TFCC tears, approaches 100% [2]. Traction applied during arthrography induces more joint space distraction than during non-arthrographic MRI, possibly due to arthrography reducing the inherent vacuum that helps maintain joint surface apposition [4].

The current traction method, using a weight and pulley device to apply traction to the wrist in the ‘Superman’ position, also distracts the elbow and shoulder in addition to the wrist. To date, no studies have investigated the prevalence and change of patient wrist pain during wrist MRI involving traction. This is relevant to institutions either currently using wrist traction or considering applying wrist traction in the future.

This study was designed to compare the level of patient pain experienced in the non-arthrographic wrist MRI without traction to that in the non-arthrographic wrist MRI with traction. Factors contributing to pain in the wrist and other body areas in wrist MRI with or without traction, as well as the effectiveness of wrist distraction, were also evaluated.

## Materials and methods

This prospective study was approved by the local Clinical Research Ethics Committee of our institution. Signed informed consent from all patients was obtained before the MRI examination.

### Patient selection

**Fig. 1:**
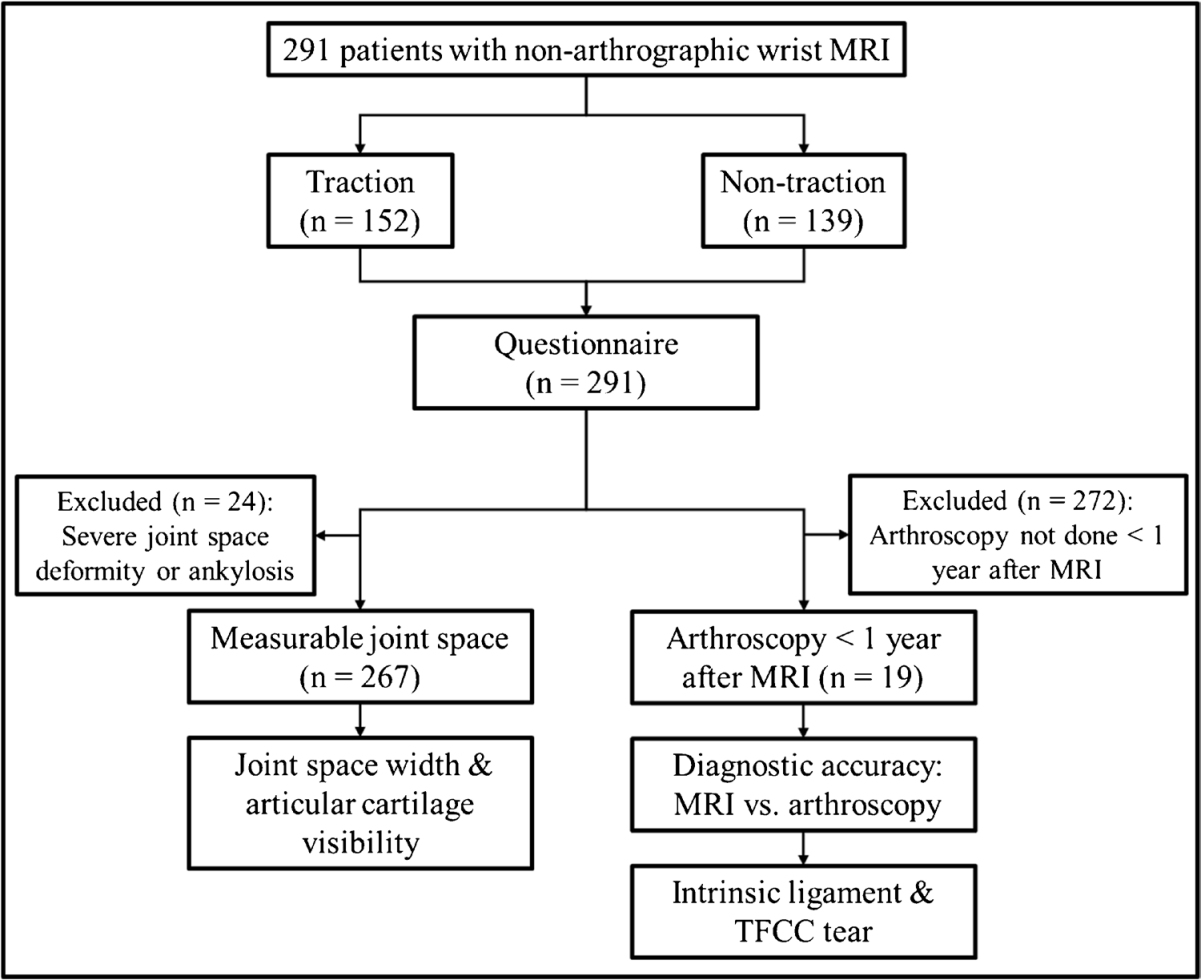
Study flowchart. TFCC, triangular fibrocartilage complex

Two hundred and ninety-one patients (109 males, 182 females; mean age, 45.8 years; range, 12–85 years) referred for non-arthrographic wrist MRI between November 2019 and September 2024 were prospectively enrolled. Of these 291 patients, 152 (52%) patients (64 males, 88 females; mean age, 42.9 years; range, 14–85 years) underwent wrist MRI with traction between September 2019 and February 2022. To compare the level of pain experienced in patients with traction and without traction, 139 (48%) subsequent patients (45 males, 94 females; mean age, 48.8 years; range, 12–78 years) underwent wrist MRI examination without traction between February 2022 and September 2024. Twelve (8%) of 152 patients in the traction group and 7 (5%) of 139 patients in the non-traction group had subsequent wrist arthroscopy within one year of wrist MRI examination. The study flowchart is shown in Fig. 1. Indications for MRI examination were categorized into three subgroups based on the information provided on the MRI request form: (1) wrist trauma; (2) arthritis, including osteoarthritis and rheumatoid arthritis; and (3) other suspected wrist pathologies, such as tenosynovitis, tendinosis, ganglion cyst, and non-specific wrist pain.

### MR imaging protocol

**Fig. 2:**
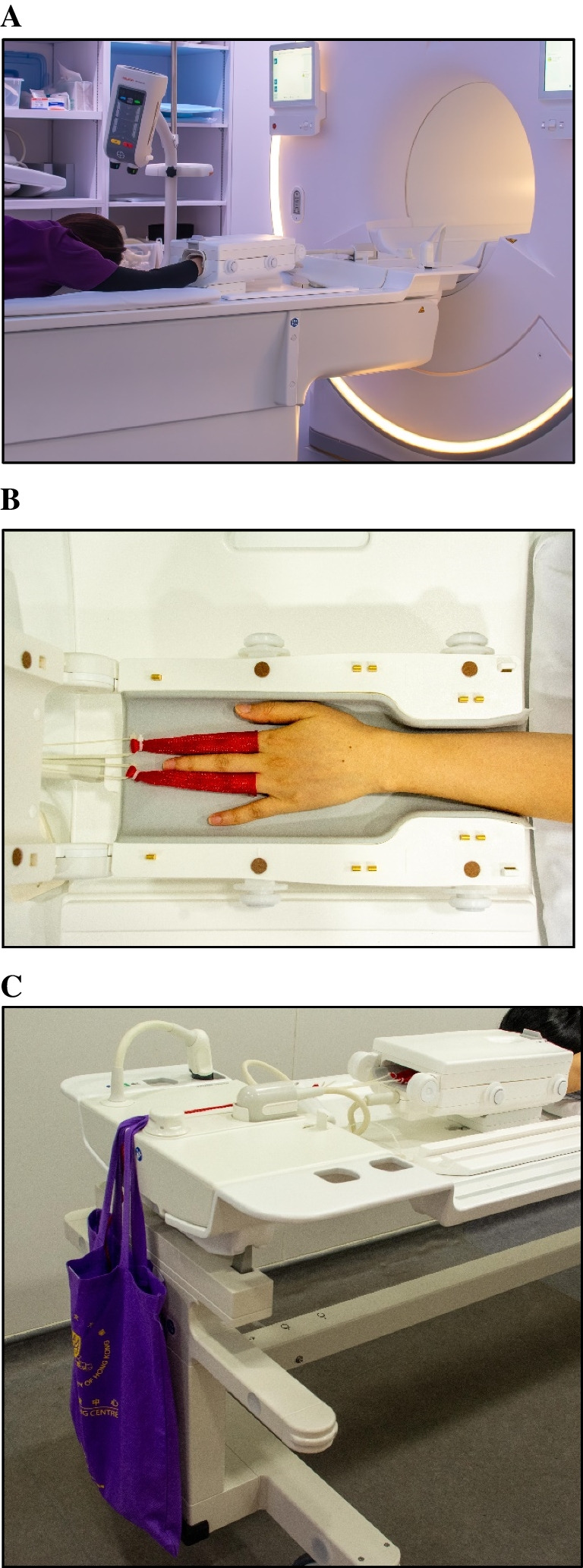
Patient positioning for wrist MRI with traction. (**A**) Patient lies in a ‘Superman’ position, with (**B**) the index and ring fingers entrapped in Chinese finger traps, (**C**) connected to suspended weights via a non-elastic cord

All MRI examinations were performed on two 3.0 T MR whole-body systems (Philips Achieva™ TX-series; Philips Ingenia Elition X, Philips Medical Systems, Best, Netherlands) utilizing a dedicated eight-channel wrist coil array (SENSE wrist coil 8; Philips Medical Systems). MRI protocol is listed in Table 1. All patients were examined in a prone position with the affected wrist fully extended above the head in a ‘Superman’ position (Fig. 2A). Traction was applied to the index and ring fingers using Chinese finger traps connected via a pulley and cable system and non-elastic cord to suspended weights (7 kg for males and 5 kg for females) (Fig. 2B and C) [4]. MRI examination time for all patients was less than 30 min, with traction application taking less than 5 min. All patients successfully completed the MRI examinations.

**Table 1:** MRI sequences of non-arthrographic wrist MRI with or without traction

MRI sequence	Plane	Slice thickness (mm)	TR (ms)	TE (ms)	Flip angle (°)	Matrix
Fat-sat PD-W	Axial	3	2496	38	90	512 × 512
Fat-sat PD-W	Coronal	2	1811	34	90	256 × 256
T1-W	Coronal	2	561	16	90	512 × 512
Fat-sat T2-W	Sagittal	2	4211	70	90	512 × 512
3D MERGE	Coronal	2	60	13	90	512 × 512

Fat-sat, fat-saturated; PD-W, proton density-weighted; T1-W, T1-weighted; T2-W, T2-weighted

### Pain severity questionnaire

All 291 patients completed an in-house-designed questionnaire within 15 min of completing the MRI examination. Utilizing a ten-point visual analogue scale (VAS) [5], this questionnaire was designed to assess severity of wrist pain before, during, and 10 min after MRI examination completion. Additional symptoms were also addressed, including overall severity and location (shoulder, neck, arm, elbow, and others) of non-wrist pain. Questionnaire completion was supervised by a research assistant. Patients were categorized into two groups: those experiencing increased wrist pain and those without such an increase, depending on whether wrist pain increased or not compared to that present prior to MRI examination.

### MR image analysis

Two radiologists, with more than 5 years of experience reporting MRI examinations, evaluated all MRI data by consensus on a PACS workstation (Carestream solution version 11.0, Carestream Health). Joint space width and articular cartilage visibility were initially evaluated in 291 wrists from 291 patients, including 129 left and 162 right wrists. Patients with severe joint space deformity or ankylosis, where the radiocarpal or intercarpal joints were unmeasurable, were excluded from MR image analysis. In the traction group, 141(93%) of 152 wrists were evaluated, 11 (7%) wrists were excluded due to unmeasurable joints. In the non-traction group, 126 (91%) of 139 wrists were evaluated, 13 (9%) wrists were excluded due to unmeasurable joints.

### Joint space width

Minimal joint space width was defined as the shortest distance between opposing cortical bone surface [3, 6]. Following zooming and contrast adjustment to optimise image clarity, minimal joint space width at five (radioscaphoid, radiolunate, scaphocapitate, lunocapitate, and triquetrohamate) radiocarpal and intercarpal joints was measured on proton-density weighted fat-saturated (PDFS) coronal images using electronic calipers to the nearest 0.1 mm. Average joint space width of the wrist joint was defined as the mean of the joint space width at all five investigated radiocarpal and intercarpal joints.

### Articular cartilage visibility

As previous studies have shown that traction improves intrinsic ligament and TFCC visibility compared to non-traction MRI, articular cartilage visibility alone was assessed in this study [2, 3, 7]. Cartilage surface visibility of the radioscaphoid, radiolunate, scaphocapitate, lunocapitate, and triquetrohamate joints was assessed on a 3-point scale on PDFS coronal images, separately for the traction and non-traction groups. “Good” visibility was defined as more than 75% of the opposing articular cartilage outline being clearly visible; “poor” visibility was when less than 25% visibility of the opposing articular cartilage outline was clearly visible; and “moderate” visibility was when clear visibility of the opposing articular cartilage outline was between 25% and 75% [8].

### Reference standard: diagnostic accuracy between MRI and arthroscopy

For patients who underwent wrist arthroscopy within one year after MRI examination, findings on MRI were compared with those on arthroscopy for full thickness scapholunate and lunotriquetral ligament tears as well as TFCC tears. Tears in the membranous portion of scapholunate and lunotriquetral ligaments were assessed, as well as tears in the articular disc or lamina of the TFCC. The diagnostic accuracy of each intrinsic ligament or TFCC tear in the two groups was evaluated, and overall diagnostic accuracy of intrinsic ligaments and TFCC tears was compared between traction and non-traction groups.

### Statistical analysis

All analyses were performed using Microsoft SPSS software, version 22.0. Quantitative data was presented as median (interquartile range). Mann–Whitney U test was used to compare differences in age, VAS pain scores, and joint space width between the traction and non-traction groups, respectively. The Chi-square test was used to compare the differences in gender, laterality of wrist, clinical indication for wrist MRI, location of non-wrist pain, and overall diagnostic accuracy of intrinsic ligaments and TFCC tears between traction and non-traction groups. Wilcoxon rank sum test was used to compare the articular cartilage visibility at the investigated radiocarpal and intercarpal joints between the traction and non-traction groups. Bootstrap method was used to compare the mean difference in average joint space width between traction and non-traction groups. Multivariate binary regression analysis was applied to investigate the potential risk factors for increased wrist pain in patients undergoing traction and non-traction wrist MRI. A two-sided *p* value < 0.05 was considered statistically significant.

## Results

### Traction versus non-traction groups

Traction group patients were younger than non-traction group patients (*p* = 0.002) (Table 2). Fewer traction group patients had ‘arthritis’ as an indication for wrist MRI than non-traction group (*p* = 0.002) (Table 2). No significant difference in gender (*p* = 0.087) (Table 2) or laterality of the wrists (*p* = 0.393) was observed between the traction and non-traction groups.

**Table 2:** Characteristics of traction and non-traction group patients

Patient	Traction (*n* = 152)	Non-traction (*n* = 139)	*p* value
Age, years, M(Q)	41 (29)	50 (24)	0.002*
Gender			
Female, n. (%)	88 (58%)	94 (68%)	0.087
Male, n. (%)	64 (42%)	45 (32%)	
Indication for wrist MRI			0.002*
Group 1, n. (%)	96 (63%)	62 (45%)	
Group 2, n. (%)	30 (20%)	52 (37%)	
Group 3, n. (%)	26 (17%)	25 (18%)	
Wrist pain			< 0.001*
Non-increased, n. (%)	95 (62%)	115 (83%)	
Increased, n. (%)	57 (38%)	24 (17%)	
Increased wrist VAS pain score, M(Q)	2(3)	1(2)	0.085
Non-wrist pain			
Prevalence, n. (%)	67(44%)	53(38%)	0.060
Location			0.436
* Neck*, n. (%)	29 (43%)	24 (45%)	
*Shoulder*, n. (%)	36 (54%)	27 (51%)	
*Arm*, n. (%)	10 (15%)	15 (28%)	
*Elbow*, n. (%)	14 (21%)	8 (15%)	
*Side of body*, n. (%)	5 (8%)	7 (13%)	
Non-wrist VAS pain score, M(Q)	5(3)	5(4)	0.500

Group 1, wrist trauma; Group 2, arthritis; Group 3, other suspected wrist pathologies. VAS, visual analogue scale. (*p value < 0.05)

### Pain severity questionnaire

**Fig. 3:**
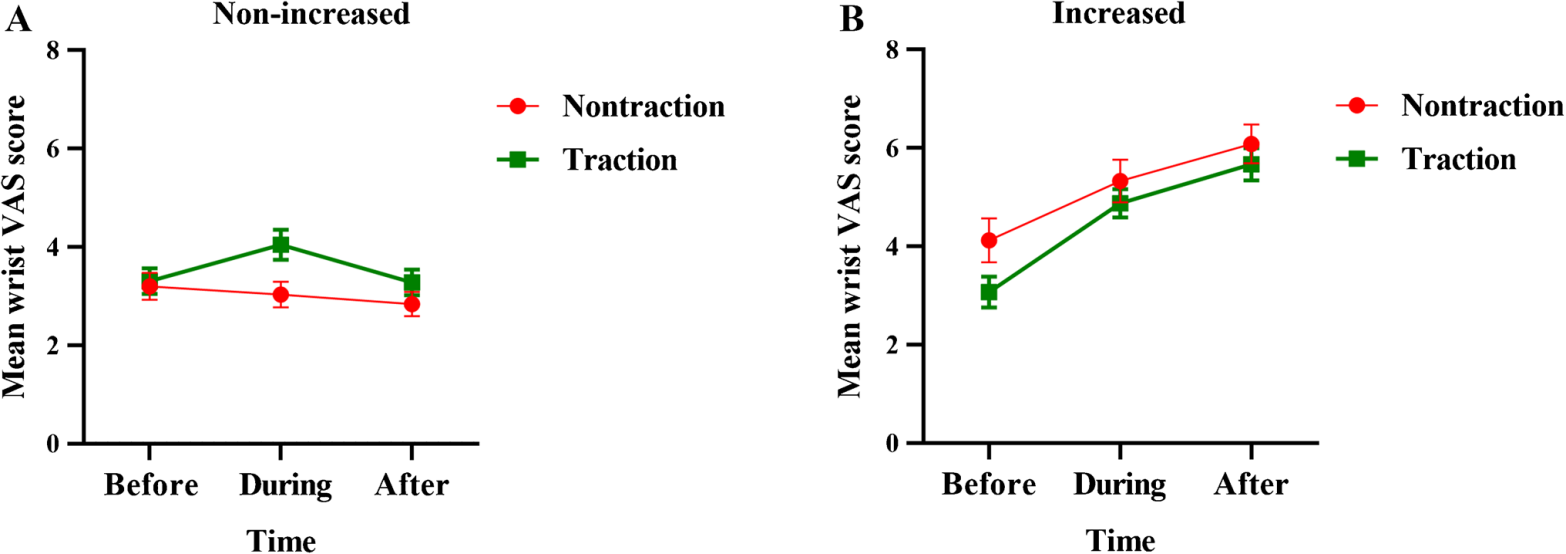
Changes of mean wrist VAS pain score over time. These line graph with error bars illustrate the changes of mean wrist Visual Analogue Scale (VAS) pain scores for traction and non-traction groups over three time points: Before, During, and After the wrist MRI. (3A) In the non-increased wrist pain subgroup, both groups did not show a significant increase in mean wrist VAS score after wrist MRI compared to that prior to wrist MRI. (3B) in the increased wrist pain subgroup, traction group patients experienced relatively rapid increase in mean wrist VAS pain score after initial application of wrist traction. Thereafter, the traction and non-traction group shared similar increasing pattern of wrist pain

95 (62%) of the 152 patients with traction and 115 (83%) of the 139 patients without traction did not report an increased VAS pain score in the wrist after the wrist MRI examination (Fig. 3A). Fifty-seven (38%) of the 152 traction group patients had increased wrist pain during MRI examination compared to 24 (17%) of the 139 non-traction group patients (*p* < 0.001), with the average increase in VAS score being 3 points (range, 1 ~ 7) for the traction group and 2 points (range, 1 ~ 6) for the non-traction group (Fig. 3B). Comparing wrist pain before MRI with during MRI and then after MRI, pain in the traction group tended to increase more steeply initially and then level off to a level what was similar to those patients without traction that also had an increase in wrist pain with MRI (Fig. 3B). Multivariate regression analysis showed that traction was the most significant risk factor for increased wrist pain during MRI examination (Table 3).

**Table 3:** Multivariate logistic analysis of potential risk factors for increased wrist pain in traction and non-traction patients

Variable	β	S.E	OR (95% CI)	*p* value
Traction	0.935	0.288	2.548(1.448, 4.482)	0.001*
Age	−0.013	0.009	0.987(0.970, 1.005)	0.161
Gender	−0.175	0.287	0.839(0.478, 1.471)	0.540
Indication for wrist MRI				
Group 1			1	0.404
Group 2	−0.447	0.366	0.639(0.312, 1.311)	0.222
Group 3	−0.319	0.376	0.727(0.348, 1.518)	0.396
Constant	−0.694	0.489	0.500	0.156

Hosmer and Lemeshow Test: *p* = 0.721. (*p value < 0.05)

Group 1, wrist trauma; Group 2, arthritis; Group 3, other suspected wrist pathologies

A comparable (*p* = 0.060) number of traction group patients (67 (44%) of 152 patients) and non-traction group patients (53 (38%) of 139 patients) reported non-wrist pain during MR examination with an average VAS pain score increase of 5 points (range 1–10 points) (Table 2). For non-wrist pain, the most common sites were the shoulder and neck (Table 2). There was no significant difference in reported location and VAS pain score of non-wrist pain between traction and non-traction groups (all *p* values > 0.05). In addition, 12 (8%) of the 152 patients in the traction group experienced finger pain due to finger distraction.

### Joint space width

**Fig. 4:**
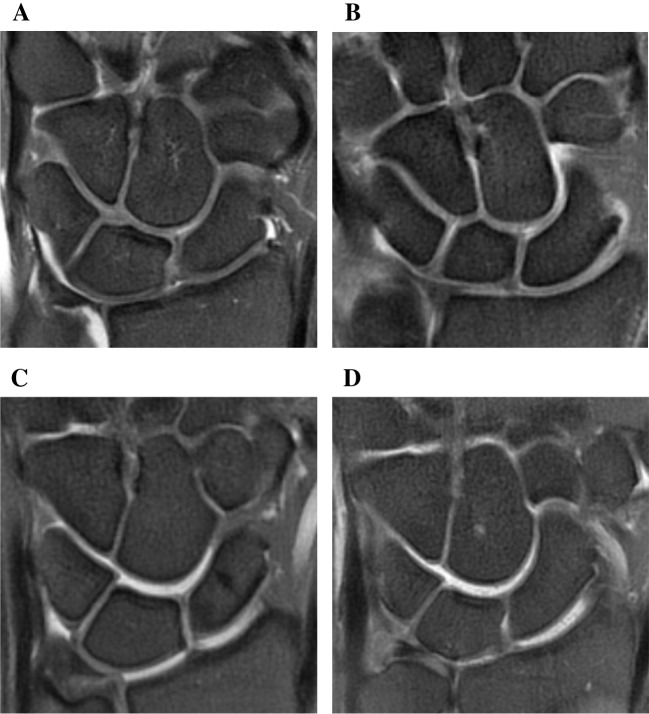
PDFS coronal left wrist MR images: (**A**) 27- and (**B**) 44-year-old female wrists without traction, (**C**) 28-year-old female and (**D**) 41-year-old male wrists with traction. Joint space width was increased in the radioscaphoid, radiolunate, scaphocapitate, lunocapitate, and triquetrohamate joints after traction

Average joint space width of the investigated radiocarpal and intercarpal joints was significantly wider (0.6 mm, 95% CI = [0.5, 0.7]) in the traction group than the non-traction group (all *p* -values < 0.001) (Table 4, Fig. 4).

**Table 4:** Comparison of joint space width and articular cartilage visibility in traction and non-traction groups

Joints	Joint space width(mm)			Articular cartilage visibility						
	Traction (*n* = 141)	Non-traction (*n* = 126)	*p* value	Traction (*n* = 141)			Non-traction (*n* = 126)			*p* value
				Poor	Moderate	Good	Poor	Moderate	Good	
Radioscaphoid	1.7 (0.8)	1.1 (0.5)	< 0.001*	24(17%)	25(18%)	92(65%)	75(60%)	35(28%)	16(13%)	< 0.001*
Radiolunate	1.8 (0.6)	1.2 (0.6)	< 0.001*	79(56%)	24(17%)	38(27%)	124(98%)	2 (2%)	0 (0%)	< 0.001*
Scaphocapitate	1.6 (0.6)	1.3 (0.5)	< 0.001*	42(30%)	56(40%)	43(30%)	87(69%)	36(29%)	3 (2%)	< 0.001*
Lunocapitate	1.7 (0.9)	1.1 (0.5)	< 0.001*	67(48%)	6 (4%)	68(48%)	123(97%)	1(1%)	2 (2%)	< 0.001*
Triquetrohamate	1.4 (0.5)	1.0 (0.4)	< 0.001*	71(50%)	44(31%)	26(18%)	112(89%)	13(10%)	1 (1%)	< 0.001*

Data are presented as M(Q) or n. (%). (*p value < 0.05)

### Articular cartilage visibility

**Fig. 5:**
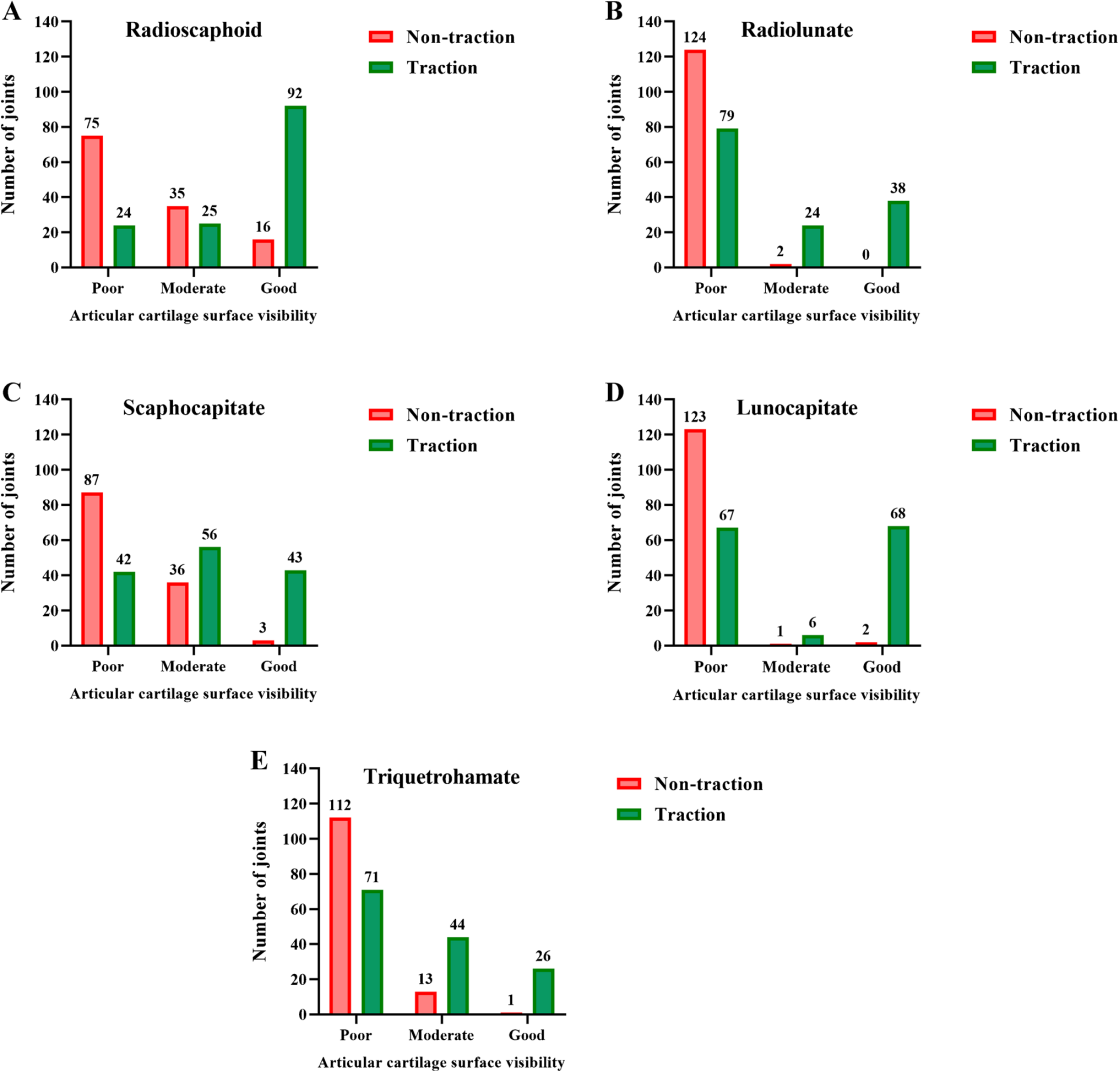
Comparison of articular cartilage visibility in non-traction and traction groups (**A**-**E**). Compared to non-traction group, “moderate” to “good” visibility of articular cartilage were observed at more than half of the radiocarpal and intercarpal joints in the traction group

Articular cartilage visibility was better in the traction group than the non-traction group (all *p* values < 0.001, Table 4, Fig. 5). On average, “moderate’’ or “good” visibility was observed in 60% (85/141) of patients in the traction group and 17% (22/126) of patients in the non-traction group. In the non-traction group, approximately 90% of the radiolunate, lunocapitate and triquetrohamate joints were graded as having “poor” visibility, whereas around 50% of these joints in the traction group were graded at least as “moderate” in visibility.

### Diagnostic accuracy for traction and non-traction MRI

Average time interval between wrist MRI and arthroscopy was 169 (188) days in the traction group and 66 (65) days in the non-traction group. In the 12 traction cases, arthroscopy identified 9 TFCC tears and 2 scapholunate ligament tears, while in the 7 non-traction cases, arthroscopy revealed 3 TFCC tears and 1 scapholunate ligament tear. Arthroscopic examination of the wrist in both groups did not reveal any lunotriquetral ligament tear. Wrist MRI in the traction group did not identify one TFCC tear, while in the non-traction group, MRI did not identify one scapholunate ligament tear and misdiagnosed one TFCC tear and one lunotriquetral ligament tear. Diagnostic accuracy of MRI for scapholunate ligament (100%), lunotriquetral ligament (100%), and TFCC (91.7%) tears in the traction group tended to be better than that in the non-traction group (85.7%, 85.7%, and 85.7%, respectively), though these differences did not reach statistical significance (*p* = 0.136).

## Discussion

Wrist MRI can be performed with or without arthrography under traction. Traction effectively separates closely opposed articular cartilage margins, thereby enhancing the visualization of the articular cartilage, intrinsic ligaments and TFCC, compared to non-traction studies [2].

More (38%) patients in the traction group than the non-traction group (17%) experienced an increase in wrist pain after MRI examination. Although a greater number of patients in the traction group reported increased wrist pain after MRI examination, the average increase in wrist pain during traction MRI compared to non-traction MRI was only 1 VAS point. The prevalence and severity of non-wrist pain were comparable between the traction and non-traction groups, suggesting that the non-wrist pain experienced during MRI examination is most likely due to the ‘Superman’ positioning rather than any traction effect. Other than a mild increase in wrist pain, no complications associated with wrist traction were observed. Similarly, no complications of wrist traction have been reported in the 55 patients from other institutions who underwent wrist traction to date [3, 6, 7].

Compared to the non-traction group, traction resulted in an increase in joint space width across all the radiocarpal and intercarpal joints subjected to distraction, with the most pronounced effect observed at the radioscaphoid joint (mean increase of 0.8 mm) and the least at the triquetrohamate joint (mean increase of 0.3 mm). Considerable individual variation in the degree of joint space widening that occurs with wrist traction likely relates to intrinsic ligament laxity or muscle tension[9]. Although the degree of increased wrist joint distraction afforded by traction was minimal, this slight separation is adequate to improve the visibility of cartilage surface, as even minimal separation of contacting cartilage interface can enhance the visibility of articular cartilage surface [4, 6]. With traction, moderate to good visibility of articular cartilage surface was observed in more than half of the joints, with a noticeable improvement in cartilage visibility evident at the radiolunate, lunocapitate and triquetrohamate joints.

A more widespread adoption of wrist traction during wrist MRI is possibly limited by the complex setup process, non-standardization of the weights used and the possibility of increased patient discomfort. Efforts should be directed towards developing a method that provides wrist traction without necessitating the ‘Superman’ position. Such a method would preserve the enhanced visibility of wrist structures that achieved through traction while mitigating the non-wrist pain associated with the ‘Superman’ position, thereby potentially offsetting the increased pain experienced with traction.

There were some limitations in this study. First, all patients in the study underwent either a traction or a non-traction MRI, with no patient undergoing both procedures, thus precluding a direct comparison of the change in VAS pain scores and the effect of joint distraction on the same patient. Second, the patients answered the questionnaire retrospectively at a single time point after completion of wrist MRI, which may have introduced bias in the severity of pain ratings before or during the MRI examination. It would have been preferable to assess patients’ pain levels three times, namely before, immediately after MRI examination, and 10 min after the MRI examination, rather than collectively rating all three pain levels approximately 15 min following completion of the MRI examination. Third, similar to previous studies [2, 3, 6], only a very small number of patients underwent subsequent wrist arthroscopy within one year of MRI examination. Therefore, while we demonstrated improved visibility of articular cartilage and previous studies have shown that traction enhances intrinsic ligament and TFCC visibility, no significant improvement in diagnostic accuracy was observed. Further studies with a larger number of patients, including those who undergo both traction and non-traction MRI followed by arthroscopy, are necessary to unequivocally demonstrate that traction does improve diagnostic accuracy. Fourth, although we regularly perform traction MR imaging of the wrist, this is not standard in most institutions, and this was a single institutional study. Experience with applying wrist traction and its effect on patient pain may not be applicable to other centers. Finally, it was not possible to remove selection bias when measuring joint space width or assessing cartilage visibility, as the traction effect was apparent on the MR images being evaluated.

In conclusion, direct traction MR imaging of the wrist increases joint space width and improves articular cartilage visibility. Traction does lead to a slight increase in wrist pain during MRI examination. No other complications were encountered.

## Supplementary Information

Below is the link to the electronic supplementary material.

Supplementary file1 (DOCX 21 KB)

## Data Availability

The data that support the findings of this study are available on request from the corresponding author, Prof. James F. Griffith. The data is not publicly available due to laws and regulations in line with the Personal Data (Privacy) Ordinance, Cap 486, Hong Kong.
