# Supplementary material for: Direct traction MR imaging of the wrist: practical experience
Source: Skeletal Radiol. 2024 Dec 4;54(7):1407–15. doi: 10.1007/s00256-024-04842-w (PMC12078367; doi:10.1007/s00256-024-04842-w)
Supplement: Supplementary file 1 — Supplementary file1 (DOCX 21 KB) [file 256_2024_4842_MOESM1_ESM.docx]

**Supplementary materials**

Table 1. Characteristics of traction and non-traction group patients with increased wrist pain.

| **Increased**  **VAS score** | | | **Traction**  (n. = 57) | **Non-traction**  (n. = 24) | **p value** | |
| --- | --- | --- | --- | --- | --- | --- |
| **Age**, years, M(Q) | | | 34 (34) | 47 (21) | 0.073 |  |
| **Gender** |  |  |  |  | 0.274 |  |
| Female, n. (%) | | | 33(57.9%) | 17(70.8%) |  |  |
| Male, n. (%) | | | 24(42.1%) | 7 (29.2%) |  |  |
| **Indication for wrist MRI** | | | | | 0.469 | |
| Group 1, n. (%) | | | 36 (63.2%) | 17 (70.8%) |  |  |
| Group 2, n. (%) | | | 10 (17.5%) | 5 (20.8%) |  |  |
| Group 3, n. (%) | | | 11 (19.3%) | 2 (8.3%) |  |  |

Group 1, wrist trauma; Group 2, arthritis; Group 3, other suspected wrist pathology. Data**^** are presented as median (interquartile range).

Table 2. Characteristics of traction and non-traction group patients with subsequent arthroscopy within 1 year after MRI.

| **Characteristic** | | | **Traction**  (n. = 12) | **Non-traction**  (n. = 7) | **p value** | |
| --- | --- | --- | --- | --- | --- | --- |
| **Age**, years, M(Q) | | | 44.5(31) | 35(27) | 0.330 |  |
| **Gender** |  |  |  |  |  |  |
| Female, n. (%) | | | 5(41.7%) | 4(57.1%) | 0.650 |  |
| Male, n. (%) | | | 7(58.3%) | 3(42.9%) |  |  |
| **Indication for wrist MRI** | | | | | 0.405 | |
| Group 1, n. (%) | | | 10 (83.3%) | 5(71.4%) |  |  |
| Group 2, n. (%) | | | 2 (16.7%) | 1(14.3%) |  |  |
| Group 3, n. (%) | | | 0 (0.0%) | 1(14.3%) |  |  |

Group 1, wrist trauma; Group 2, arthritis; Group 3, other suspected wrist pathology. Data**^** are presented as median (interquartile range).
